# Densely Deployed Indoor Massive MIMO Experiment: From Small Cells to Spectrum Sharing to Cooperation

**DOI:** 10.3390/s21134346

**Published:** 2021-06-25

**Authors:** Andrea P. Guevara, Sofie Pollin

**Affiliations:** Department of Electrical Engineering, KU Leuven, 3001 Leuven, Belgium; spollin@esat.kuleuven.be

**Keywords:** massive MIMO, spectral efficiency, spectrum sharing, infrastructure sharing, infrastructure cooperation

## Abstract

Massive MIMO is a key 5G technology that achieves high spectral efficiency and capacity by significantly increasing the number of antennas per cell. Furthermore, due to precoding, massive MIMO allows co-channel interference cancellation across cells. In this work, based on experimental channel data for an indoor scenario, we analyse the impact of inter and intra-cell interference suppression in terms of spectral efficiency, capacity, user fairness and computational cost for three simulated systems under different cooperation levels. The first scenario assumes a cooperative case where eight neighbouring cells share the spectrum and infrastructure. This scenario provides the highest system performance; however, user fairness is achieved only when there is inter and intra-cell interference suppression. The second scenario considers eight cells that only share the spectrum; with full intra-cell and inter-cell interference cancellation, it is possible to achieve 32% of the optimal capacity with 20% of the computational cost in each distributed CPU, although the total computational cost per system is the highest. The third scenario considers eight independent cells operating in different frequency bands; in this case, intra-cell interference suppression leads to higher spectral efficiency compared to the cooperative case without intra-cell interference suppression.

## 1. Introduction

5G and beyond will combine various applications with a wide range of tight Quality-of-Service (QoS) requirements. One of the technologies that can fulfil those diverse requirements is massive multiple-input multiple-output (MIMO). In massive MIMO systems, a base station (BS) with multiple antennas can serve a large number of users at the same time and with the same bandwidth resources, enabling high spectral efficiency and capacity. System capacity improvements are only limited by pilot contamination and channel estimation, and as a result, interference during data transmission can be neglected when a large number of antennas is assumed [1].

In reality, the number of antennas is limited. To maximise coverage and favourable propagation conditions, while minimising the impact of shadowing, distributing the antennas over the targeted area is meaningful [2,3,4,5,6,7,8]. When multiple distributed antennas are processed jointly and coherently, we call this a distributed massive MIMO system. Nonetheless, a distributed massive MIMO system requires a higher load of data transmission in the back-haul and strict synchronisation between the multiple antennas to combine the transmitted and received signals coherently. This leads to a higher cost in infrastructure expenditure for a single massive MIMO system that intends to deploy a large number of antennas in a given area. Thus multiple massive MIMO systems can cooperate by sharing access to their infrastructure to generate an extensive system with a vast number of antennas dispersed across a particular area.

The infrastructure cooperation brings both technical improvements and economic incentives. In [9], the authors analyse the cooperation between two or more operators, which lend their network resources to third-party networks under different levels of agreements. This work shows a reduction in operating and capital expenses, which leads to a favourable option from the economic point of view. In [10], the authors also analyse the full cooperation in terms of spectrum and infrastructure, evaluating the performance between multiple mmWave networks through simulations. Likewise, in [11], the authors carried out an outdoor massive MIMO experiment to collect data to create two virtual cells. Here, the performance in terms of spectrum efficiency, capacity and coverage area is analysed. In the same way as the previous works, reference [11] also demonstrates that cooperation among systems leads not only to a monetary reward but also increases the performance of the stakeholders involved.

Cooperation between systems can also be done by spectrum sharing when there are multiple neighbouring cells, which could reuse the same spectrum as this resource still remains scarce. Nevertheless, spectrum cooperation or spectrum sharing between multiple networks increases interference in the system.

One of the key characteristics of massive MIMO is co-channel interference suppression that is achieved with the combining vector during up-link transmission. Multiple techniques suppress interference in different forms, such as Minimum Mean Squared Ratio (MMSE) [12], which suppresses all interference and reduces the noise; this technique is named as optimal. However, it requires a full wireless channel knowledge, which is unrealistic in real scenarios unless some approximations are presented beforehand, which requires a huge computational cost. On the other hand, Zero-Forcing (ZF) [13] relies on the estimated channel, and this combining vector increases the desired signal power while suppressing interference, but this technique works in a higher signal-to-noise ratio (SNR) regime.

The state-of-the-art considers a ZF combining vector in single cells, which suppress interference of other users within this cell named intra-cell interference; however, the selection of these interferences may vary. In [14], the authors propose a ZF combining vector, which suppresses all the interference within the same and neighbouring cells. However, in scenarios where the interference contribution between users varies due to path-loss, a selection of strong interferences to be suppressed can be applied to reduce computational cost and back-haul data when the systems are distributed or work under an infrastructure sharing scheme. Thus, in [15,16], the users to cancel are those served by the same base station. In [14,17,18], those users are selected based on the interference power towards the desired user. Finally, in [19], the suppression is carried out towards the eigenvectors related to the wireless channel rather than the channels themselves.

In this paper, we show, using experiments, that there is still a significant inter-cell co-channel interference even when considering massive MIMO with a large number of antennas. Thus, we analyse the impact of inter and intra-cell interference suppression for two network metrics: spectral efficiency and capacity under the analysis of three indoor scenarios. Each of those scenarios has different levels of cooperation in terms of spectrum and infrastructure sharing.

The paper is organised as follows: Section 2 describes our system model and a set of combining vectors given in the literature and how they relate to the spectral efficiency and capacity of the system. Section 4 describes the experiment carried out to obtain the results discussed in Section 6. To finalise, Section 8 draws the main conclusions.

The following notation is used throughout the paper: $x\in C^{M}$ represents an M complex vector, $X\in C^{M\times N}$ is an M×N complex matrix. $x^{H}$ and $x^{T}$ states a transpose and conjugate-transpose of vector x. The $L_{2}$ norm for vector **x** is given as ∥x∥ and |y| for the absolute value of a scalar y. E{x} is the expectation of **x**.

## 2. System Model

In a multi-cell massive MIMO system with *L* cells, each cell *j* has $K_{j}$ users that are associated with a single BS*j* with $M_{j}$ antennas. The wireless channel between any user $k\in \{1,\ldots ,K_{j}\}$ and BS within cell *j* is $h_{\mathrm{jk}}^{j}\in C^{M}$, with a user transmit power of $p_{\mathrm{jk}}$. The channel between user *i* in cell *l* and BS in cell *j* is $h_{\mathrm{li}}^{j}\in C^{M}$ and the channel gain (β) is defined as:

(1) $$\beta_{\mathrm{li}}^{j}=E{\{||h}_{\mathrm{li}}^{j}{\left|\right|}^{2}\}.$$

The channel of each user *i* is associated with an individual spatial correlation matrix $R_{\mathrm{li}}^{j}\in C^{M\times M}$ given by:

(2) $$R_{\mathrm{li}}^{j}=E\left\{h_{\mathrm{li}}^{j}{h_{\mathrm{li}}^{j}}^{H}\right\}.$$

It is worth mentioning that in this work, we consider a perfectly known channel correlation matrix for simplicity. However, (2) can be replaced by imperfect channel covariance information, as discussed in [20].

### 2.1. Signal to Interference Approximation

**Figure 1:**
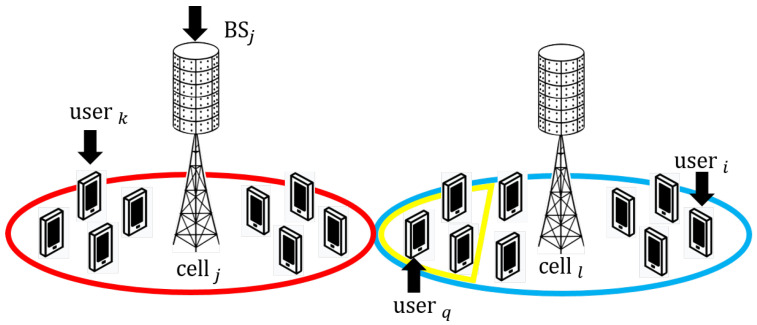
Intra-cell interference is caused during up-link transmission between user *k* and any other user in cell *j* (red circles). Inter-cell interference is caused by users in the neighbouring cell (blue circle). Users within the yellow circle cause dominant inter-cell interference.

To illustrate intra-cell and inter-cell interference, Figure 1 shows a two-cell massive MIMO system. All users within the red circle are served by BS *j* and users inside the blue circle by BS *l*. The generic Signal-to-Interference Ratio (SIR) expression between BS *j* and user *k* and any other interfering user *i*, in absence of precoding or receive processing, is given by:

(3) $$SIR_{k\;\mathrm{int}}^{j}=\frac{p_{\mathrm{jk}}E{\{||h}_{\mathrm{jk}}^{j}{\left|\right|}^{2}\}}{p_{i}E{\{||h}_{i}^{j}{\left|\right|}^{2}\}}=\frac{p_{\mathrm{jk}}\beta_{\mathrm{jk}}^{j}}{p_{i}\beta_{i}^{j}}\;k\neq i.$$

The term $p_{i}\beta_{i}^{j}$ represents the interference power from any other user *i* (i≠k), in the same and other cells. If this interference is caused by a user i inside the cell, it is called intra-cell interference. As those users are close to BS *j*, their interference causes a large impact during transmission, reducing the ${\mathrm{SIR}}_{i}$. When the interference is caused by any user *i* outside the analysed cell, in this case, outside the red circle, it is called inter-cell interference.

**Figure 2:**
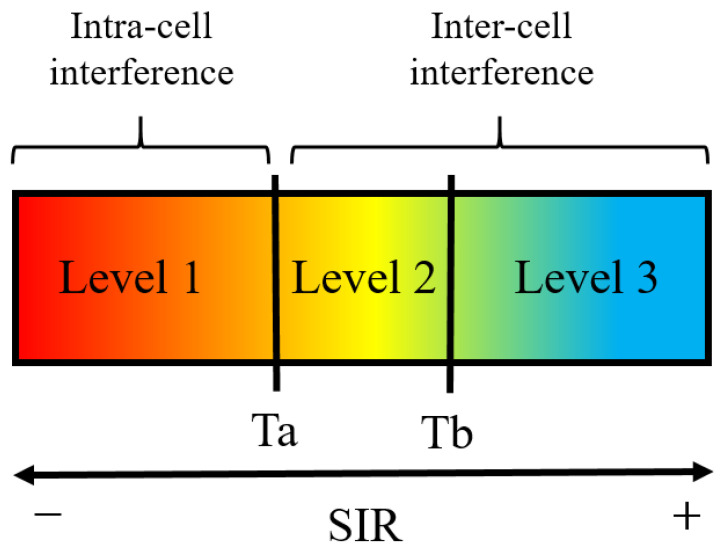
Different levels of signal-to-interference ratio (SIR) during up-link transmission between user *k* and BS *j*.

The level of interference is classified according to a SIR scale, as shown in Figure 2. Therefore, users that cause intra-cell interference to user *k*, will be allocated in Level 1, and users that cause inter-cell interference will be allocated between Level 2 and Level 3. In [21], analysis results for an experimental outdoor scenario showed that a good interference suppression could be achieved by classifying individual interference $SIR_{i}$ according to and interference threshold Tb. The analysis concludes that users with $SIR_{i}$ below the threshold do not contribute to interference and should be considered as noise.

In this work, interference suppression is analysed as part of an extended ZF (E-ZF) combining vector and compared with simple Maximum-Ratio Combining (MRC). The main goal is to understand how much harm inter-cell and intra-cell interference causes in an indoor scenario. Still, instead of relying on SIR thresholds, we propose to quantify interference as a number of dominant interfering users that are suppressed.

### 2.2. Channel Estimation

The Channel State Information (CSI) is the set of channel response realisations known by any BS. A common method to obtain CSI is MMSE channel estimation. During up-link training, an orthogonal pilot sequence $\Phi_{\mathrm{jk}}\in C^{\tau_{p}},$ with length $\tau_{p}$ ($\tau_{p}$ up-link data samples per coherence block are used to transmit a pilot sequence) is allocated to each user *k* in cell *j*. Thus, BS in cell *j* receives channel information from users within the same cell and users in all (L−1) adjacent cells, plus a noise N with element-wisely distributed as $N(0,\;\sigma_{UL}^{2})$, $N\in C^{M_{j}\times \tau_{p}}$ as $Y_{j}^{pilot}\in C^{M_{j}\times \tau_{p}}$:

(4) $$Y_{j}^{pilot}=\sum_{k=1}^{K_{j}}h_{\mathrm{jk}}^{j}\Phi_{\mathrm{jk}}^{T}+\sum_{\begin{array}{c} l=1\\ l\neq j \end{array}}^{L}\sum_{i=1}^{K_{l}}h_{\mathrm{li}}^{j}\Phi_{\mathrm{li}}^{T}+N.$$

Once the up-link signal reaches BS in cell *j*, it is multiplied by the conjugate of each pilot sequence *jk*, to obtain $y_{\mathrm{jjk}}^{pilot}\in C^{M_{j}}$:

(5) $$y_{\mathrm{jjk}}^{pilot}=Y_{j}^{pilot}\Phi_{\mathrm{jk}}^{*}=\sum_{k=1}^{K_{j}}h_{\mathrm{jk}}^{j}\Phi_{\mathrm{jk}}^{T}\Phi_{\mathrm{jk}}^{*}+\sum_{\begin{array}{c} l=1\\ l\neq j \end{array}}^{L}\sum_{i=1}^{K_{l}}h_{\mathrm{li}}^{j}\Phi_{\mathrm{li}}^{T}\Phi_{\mathrm{jk}}^{*}+N\Phi_{\mathrm{jk}}^{*}.$$

If all the pilot sequences for all users in the *L* cells are orthogonal, ($\Phi_{\mathrm{jk}}$⊥$\Phi_{\mathrm{li}}$) a non-pilot contamination scenario is considered. In the case of pilot contamination, the received signal contains a summation of desired and contaminated channels.

After pilot decorrelation in (5), an MMSE channel estimation process is carried out. Based on Theorem 3.1 from [22], the estimated channel ${\hat{h}}_{\mathrm{jk}}^{j}\in C^{M_{j}}$ is obtained as:

(6) $${\hat{h}}_{\mathrm{jk}}^{j}=\sqrt{p_{\mathrm{jk}}}R_{\mathrm{jk}}^{j}\Psi_{\mathrm{jk}}y_{\mathrm{jk}}^{j}.$$

where the MMSE term $\Psi_{\mathrm{jk}}\in C^{M_{j}\times M_{j}}$ is given as:

(7) $$\Psi_{\mathrm{jk}}={\left(\tau_{p}\sum_{j^{\prime }k^{\prime }\in \xi_{\mathrm{jk}}}\sqrt{p_{j^{\prime }k^{\prime }}}R_{j^{\prime }k^{\prime }}+\sigma_{UL}^{2}I_{M_{j}}\right)}^{-1}.$$

$I_{M_{j}}$ is the identity squared matrix of order $M_{j}$ and $\xi_{\mathrm{jk}}$ represents the set of users in all *L* cells that use the same pilot sequence *jk* and cause pilot contamination. The noise variance ($\sigma_{UL}^{2}$) is obtained through the system signal-to-noise ratio (SNR). Therefore, the estimated channel matrix, ${\hat{H}}_{j}^{j}\in C^{M_{j}\times K_{j}}$, in cell *j* is given by:

(8) $${\hat{H}}_{j}^{j}=\left[{\hat{h}}_{j1}^{j}...{\hat{h}}_{{\mathrm{jK}}_{j}}^{j}\right].$$

Similar to (2), the correlation error matrix, $C_{\mathrm{jk}}^{j}\in C^{M_{j}\times K_{j}}$, is obtained as:

(9) $$C_{\mathrm{jk}}^{j}=E\left\{\left(h_{\mathrm{jk}}^{j}-{\hat{h}}_{\mathrm{jk}}^{j}\right){\left(h_{\mathrm{jk}}^{j}-{\hat{h}}_{\mathrm{jk}}^{j}\right)}^{H}\right\}.$$

where the channel error is represented as ${\tilde{h}}_{\mathrm{jk}}^{j}=h_{\mathrm{jk}}^{j}-{\hat{h}}_{\mathrm{jk}}^{j}$, ${\tilde{h}}_{\mathrm{jk}}^{j}\in C^{M_{j}}$.

### 2.3. Up-Link Transmission

After channel estimation and during up-link transmission, user *k* in cell *j* sends over its channel a data signal $s_{\mathrm{jk}}\in C$. All other users within cell *j* and users in other cells also transmit up-link signals simultaneously.

The signals mentioned above plus noise are combined and received by the BS in cell *j* as $y_{j}\in C^{M_{j}}$. It is worth emphasising that the up-link desired signal ($s_{\mathrm{jk}}$) will be received at the BS over the estimated channel and error channel as follows:

(10) yj=∑l=1l≠jh^jkjsjk︸Estimated channel+∑l=1l≠jh˜jkjsjk︸Channel error+∑i=1i≠kKjhjijsji︸Intra-cell interference+∑l=1l≠jL∑i=1Klhlijsli︸Inter-cell interference+∑l=1l≠jn.︸Noise

Once the received signal arrives at the BS, state-of-the-art combining vectors $\left(v_{\mathrm{jk}}\in C^{M_{j}}\right)$ estimate the desired signal of each user. Each combining vector deals differently with the signal components in (10). Thus, the Maximum-Ratio Combining (MRC) [23] increases the power of the desired signal but does not suppress either interference or noise. In the case of Zero-Forcing (ZF) [13] and Regular Zero-Forcing (RZF), intra-cell interference is nullified as well as noise when RZF is applied. The Minimum-Mean Squared Error (MMSE) [12] combining vector cancels intra-cell interference, channel errors and noise from the received signal. However, MMSE requires full knowledge of the channel estimation error, which in real scenarios is unknown.

Inspired by partial MMSE-based interference suppression in the state-of-the-art, we consider two partial ZF methods in this work. The first one suppresses inter and intra-cell interference, and the second variation partially suppresses the interference of selected dominant interferers (intra and inter-cell).

### 2.4. SINR and Up-Link Spectral Efficiency

After coupling the received signal in (10) with any combining vector $v_{\mathrm{jk}}$, Theorem 4.1 in [22] proposes an instantaneous SINR of the *jk* user (under the assumption that MMSE is used as channel estimation), as follows:

(11) $$SINR_{\mathrm{jk}}=\frac{p_{\mathrm{jk}}{\left|v_{\mathrm{jk}}^{H}{\hat{h}}_{\mathrm{jk}}^{j}\right|}^{2}}{\sum_{l=1}^{L}\sum_{\begin{array}{c} i=1\\ l\neq j\\ i\neq k \end{array}}^{K_{l}}p_{\mathrm{li}}{\left|v_{\mathrm{jk}}^{H}{\hat{h}}_{\mathrm{li}}^{j}\right|}^{2}+v_{\mathrm{jk}}^{H}\left(\sum_{l=1}^{L}\sum_{i=1}^{K}lp_{\mathrm{li}}C_{\mathrm{li}}^{j}+\sigma_{UL}^{2}I_{M}j\right)v_{\mathrm{jk}}}.$$

Using the lower bounded capacity from the ergodic up-link as in [15], the SE of user *k* in cell *j* is given by

(12) $$SE_{\mathrm{jk}}=\frac{\tau_{u}}{\tau_{c}}E\left\{log_{2}\left(1+SINR_{\mathrm{jk}}^{UL}\right)\right\}.$$

where $\tau_{u}\tau_{c}$ ($\tau_{u}$ is the up-link data samples received per coherence block, and $\tau_{c}$ is the total number of samples per coherence block) is the portion per coherence block (a coherence block is the set of sub-carriers and samples over time and frequency for which its impulse response tends to be constant [22]) used to transmit UL data.

### 2.5. Combining Vectors

Multiple combining vectors have been proposed in the state-of-the-art [22]. These rely on different types of channel state information to coherently combine the desired signals; this information could be: channel estimation error, intra-cell information, or intra- and inter-cell information, as described below:

#### 2.5.1. Maximum-Ratio Combining (MRC)

This combining vector relies only on the estimated channel matrix within the analysed cell *j* to maximise all desired signals:

(13) $$v_{\mathrm{jk}}^{jMR}={\hat{h}}_{\mathrm{jk}}^{j}.$$

This combining vector is one of the most used due to its low computational complexity.

#### 2.5.2. Extended Zero-Forcing (E-ZF)

The ZF combining vector known by the state-of-the-art makes a trade-off between suppressing the intra-cell interference while increasing the value of the desired signal. In this work, we propose an extension of the ZF combining vector, hence its name, which considers intra and inter-cell interference. The selection of the interfering users can lead to a full or partial interference suppression, and those are included in the channel matrix ${\hat{H}}^{j}$. Therefore, the E-ZF matrix applied by the BS in cell *j* is expressed as:

(14) $$V^{jE-ZF}={\hat{H}}^{j}{\left({\left({\hat{H}}^{j}\right)}^{H}{\hat{H}}^{j}\right)}^{-1}.$$

The E-ZF combining vector for user *k* in cell *j*, $v_{\mathrm{jk}}^{jE-ZF}$ is obtained as the first vector of (14). The dimensions of the complex matrix $V^{jE-ZF}$ are equal to the dimensions of ${\hat{H}}^{j}$. The channel matrix ${\hat{H}}^{j}$ is constructed in the following forms.

#### 2.5.3. Full Interference Suppression

In order to suppress all interference, the channel from the interfering users must be concatenated in a suppression matrix. As ${\hat{H}}^{j}$ represents the estimated channel matrix for intra-cell interferers, then the remaining inter-cell interference matrices must be included as follows

(15) $${\hat{H}}^{j}=\left[{\hat{h}}_{\mathrm{jk}}^{j}\;\underset{\mathrm{Intra}-\mathrm{cell}\;\mathrm{interference}}{\underset{︸}{{\hat{h}}_{1j}^{j}\cdots {\hat{h}}_{j(k-1)}^{j}\;{\hat{h}}_{j(k+1)}^{j}\cdots {\hat{h}}_{{\mathrm{jK}}_{j}}^{j}}}\;\underset{\mathrm{Inter}-\mathrm{cell}\;\mathrm{interference}}{\underset{︸}{{\hat{H}}_{1}^{j}\cdots {\hat{H}}_{j-1}^{j}\;{\hat{H}}_{j+1}^{j}\cdots {\hat{H}}_{L}^{j}}}\right].$$

Note that, in this case, ${\hat{H}}^{j}\in C^{M\times \mathrm{KL}}$.

#### 2.5.4. Partial Interference Suppression

As not all the interfering users contribute the same amount of interference, here ${\hat{H}}^{j}$ selects only a set of $Q^{j}$ users that cause a strong interference to any signal transmitted within cell *j*. The set of users $Q^{j}$ are called dominant interferes; those users can be located in any cell in the system. Let us assume that all *L* cells have the same number of *K* users, then:

(16) |Q|≤(|K|−1)|L|,

where the analysed user $k\notin Q^{j}$. In this case, the partial estimated channel matrix towards BS in cell *j* is given as

(17) $${\hat{H}}^{j}=\left[{\hat{h}}_{\mathrm{jk}}^{j}\;\underset{\mathrm{Dominant}\;\mathrm{interferes}}{\underset{︸}{{\hat{h}}_{1}^{j}\cdots {\hat{h}}_{Q^{j}}^{j}}}\right].$$

With a matrix size ${\hat{H}}^{j}\in C^{M\times (Q+1)}$.

## 3. Computational Cost

We consider the computational cost as the amount of complex multiplications and divisions required to estimate each combining vector (additions and subtractions also contribute to the computational cost. However, their contribution is small and are neglected in this work).

Lemma B.1 in [22] states that given two matrices $X\in C^{a\times b}$ and $Y\in C^{b\times c}$, the multiplication of **XY** requires *abc* complex multiplications. While the multiplication a matrix **X** by its transpose conjugate ${\mathrm{XX}}^{H}$ needs $\frac{a^{2}+a}{2}b$ complex multiplications. Lemma B.2 in [22] states that the matrix inversion using a Cholesky decomposition of a Hermitian positive semi-definite matrix $X\in C^{a\times a}$ requires $\frac{a^{3}-a}{3}$ complex multiplications.

Based on (14), the estimation of the inner complex multiplication between the channel matrix and its transpose conjugate $H^{H}H$ depends on the size of H. Thus, for a full interference suppression, this size is M×KL then $({\left(\mathrm{KL}\right)}^{2}+\mathrm{KL})M/2$ is the computational cost. For a partial interference suppression with a matrix size of M×(Q+1), $({\left(Q+1\right)}^{2}+\left(Q+1\right))M/2$ complex multiplications are needed.

The second term is the matrix inversion of the E-ZF combining vector, based on the Cholesky decomposition it requires $({\left(\mathrm{KL}\right)}^{3}-\mathrm{KL})3$ complex multiplications for the full interference suppression and $({\left(Q+1\right)}^{3}-\left(Q+1\right))3$ for the partial one. Finally, the multiplication between H and the E-ZF inverse matrix requires ${\left(\mathrm{KL}\right)}^{2}$ and ${\left(Q+1\right)}^{2}$ times for full and partial interference suppression, respectively. A summary is presented in Table 1.

The MRC vector does not require any complex multiplication as it relies only on the estimated channel.

## 4. Experiments

In this work, we evaluate the performance of the stated combining vectors under different virtual scenarios relying on data obtained in a dense indoor massive MIMO experiment with the aid of the KU Leuven Massive MIMO testbed [24].

### 4.1. Massive MIMO Experiment

**Figure 3:**
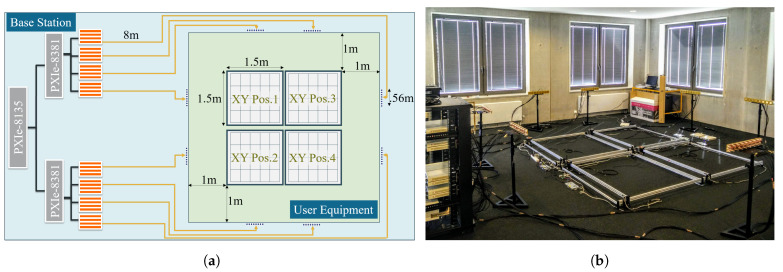
Massive MIMO LoS experimental scenario deployment for eight distributed ULA antenna array. (**a**) Experimental schema. (**b**) Real experiment.

The massive MIMO testbed comprises two main components, a base station with 64-patch antennas and a set of single-antenna user equipment. One of the unique characteristics of this testbed is the modularity of the base station antennas, which can be easily distributed. In this indoor experiment, those antennas were deployed as eight distributed linear arrays. Each of those arrays contains a set of 1×8 antennas deployed in the outskirts of the experimental area at a 1 meter distance, as shown in Figure 3.

In this experiment, we use four user’s equipment deployed on the ground. The dipole antenna of each user’s equipment is mounted on an XY automated positioner. The four positioners moved synchronously in an approximated 3 × 3 m total area. Every 25 cm in the X-axis and every 30 cm in the Y-axis the positioners moved simultaneously and stopped for 30 s, while the wireless channel was collected at the base station in real-time by the LabVIEW Communications MIMO Application Framework 1.1 [25]. The channel vector collected per position *k* is named $h_{k}^{meas}\in C^{M\times F}$, where M is the 64-patch antennas and F is the number of sub-carriers, for this setup F is 100.

During post-processing, the wireless channel is separate according to each XY position. Then, all 120 positions are treated as virtual users.

### 4.2. Data Processing

After channel collection, the experimental wireless channel ($h_{k}^{meas}$) was processed offline. The first step was the normalisation of the channel over all number of sub-carries. We normalise the channel to be able to simulate various SNR scenarios easily.

(18) $$h_{k}\left(f\right)=\frac{h_{k}^{meas}\left(f\right)}{\sqrt{\sum_{M}|\left|h_{k}^{meas}\left(f\right)\right|{|}^{2}}}.$$

**Figure 4:**
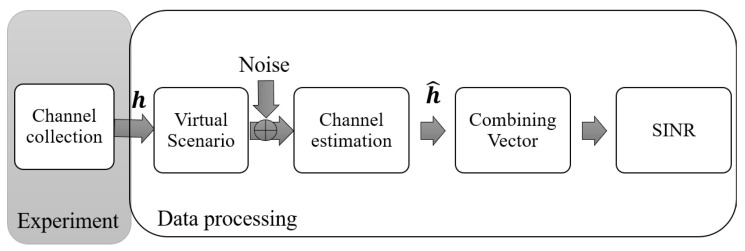
Sequence of the data collected during the ultra-dense experiment and its process offline.

The second step is the selection of the virtual scenarios and the allocation of the users to each access point, which is based on the channel gain, described in detail in Section 5. Then, the effective SNR was set to 20 dB; in this way, additional noise was added so MMSE channel estimation could be carried out. Therefore, the collected channel represents **h**, and after MMSE channel estimation, it is possible to obtain $\hat{h}$. As a fourth step, the different combining vectors are computed, concluding with the estimation of the SINR and spectral efficiency. This process is depicted in Figure 4.

#### Experimental Application

The deployment of the antennas and users in this indoor experiment simulates an automated industrial scenario. Here, each of the named virtual users represents a wireless ambient floor sensor that controls robots that move above them. The function of the robots is to move different products automatically. Thus, the main goal of the system is to offer a higher performance in real-time for indoor mobility. A more detailed explanation of this futuristic scenario is given as a proposed showcase 2: *Low Latency Industrial Communication*, as part of the “Orchestration and Reconfiguration Control Architecture” (ORCA) project [26].

## 5. Scenarios and Different Levels of Cooperation

The layout of the experiment described in Section 4 allows us to consider an eight-cell system. Thus, during data processing, we can assume that each cell has a virtual base station equipped with eight antennas. Then, from the set of 120 positions (also known as virtual users), we randomly select a set of eight different virtual users and their channel information. The users are allocated to the cells according to the following levels of cooperation in terms of spectrum and infrastructure.

**Figure 5:**
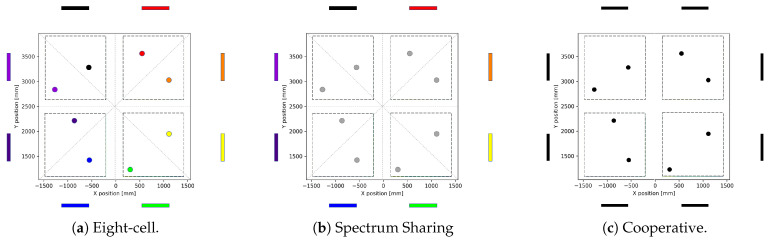
Three MIMO scenarios under different levels of cooperation. (**a**) Eight independent cells (different array and user colours) working in different frequency bands. (**b**) Eight cells sharing the same frequency band. (**c**) Eight fully synchronised cells that share spectrum and infrastructure.

We consider a system with 20 MHz bandwidth with three different proposed scenarios:

- Eight-cell scenario: In this virtual scenario, we assume eight independent cells. There is no back-haul connection between them, and each of these cells has its own CPU. Figure 5a depicts this scenario, where we have eight base stations and those antenna arrays are represented with different colours in the outskirts of the area of service. The users have the same colour as the antenna arrays that served them. We assume that each cell works at a different carrier frequency and has 2.5 MHz bandwidth; thus, for this case, there is no inter-cell interference.
- Spectrum sharing scenario: In this case, we consider eight-cells coordinated with a central CPU to allocate the users to the closest base station’s array and share information for interference suppression. However, all the cells have a local CPU for up-link and down-link transmission processing. All cells operate at the same centre frequency and have a combined bandwidth of 20 MHz; in other words, they share a spectrum. Therefore, inter-cell interference will impact the performance of each cell. In Figure 5b, we can see the eight different antennas array colours for each cell, while the colour of the users are grey as they cause inter-cell interference to each other.
- Cooperative scenario: In this case, we consider a fully cooperative distributed system, where the eight cells are perfectly synchronised in the back-haul to a single CPU. The CPU is in charge of up-link and down-link transmission data processing. This scenario can also be named as a distributed massive MIMO system where eight base stations cooperate, serving all the users simultaneously with a total bandwidth of 20 MHz, see Figure 5c. As a single cell, there is only intra-cell interference.

## 6. Performance Evaluation

Based on the experiment dataset, we created 100 different service cases by randomly selecting eight virtual users per service case. Those virtual users are allocated to the antenna array with the highest channel gain (β). Then the evaluation of the spectral efficiency and capacity is carried out according to the different levels of cell cooperation described in the previous section. The parameters per scenario based on the different levels of cooperation are presented in Table 2.

### 6.1. Impact of Interference Suppression

**Figure 6:**
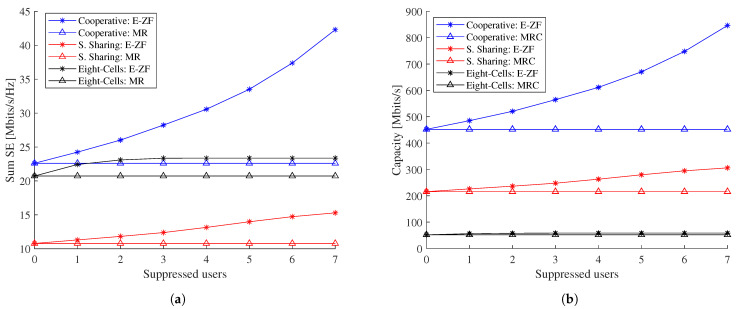
Network performance metrics vs. the numbers of suppressed interfered users for E-ZF and MRC combining vectors. The analysis includes three system configurations: Cooperative, Spectrum Sharing and Eight-Cell, scenarios. (**a**) Sum spectral efficiency per system configuration. (**b**) Total capacity per system configuration.

In Section 2.5, based on the E-ZF combining vector, we analyse how the interference can be suppressed based on selecting only a *Q* number of users with the strongest channel gain. Those results are presented in Figure 6, where we can see the impact of interference suppression in function of spectral efficiency and capacity, for the different scenarios based on levels of cooperation.

In terms of spectral efficiency, Figure 6a shows a system that shares spectrum and is perfectly coordinated (also known as cooperative scenario, blue line), which provides the highest spectral efficiency when there is no interference suppression (*Q* = 0) due to the large number of antennas that serve together to the required users. Interestingly, we can see that when the number of suppressed users increases, the SE improvements accelerate. In this case, it is noticeable that when all seven interfering users are suppressed, the spectral efficiency doubles thanks to the cooperation of 64 antennas.

When eight-cells work independently (eight-cell case, black line) and Q≥1, its E-ZF spectral efficiency value is higher than the MRC in the cooperative case. The eight-cell case has no inter-cell interference, as neighbouring cells use another frequency; therefore, based on all the multiple random user allocations, a maximum of four users are placed in the same cell, and only three intra-cell interference users can be suppressed.

The scenario with partial cooperation (eight-cells that share spectrum, red line) has the lowest spectral efficiency. In this case, with E-ZF combining vector inter and intra-cell interference is suppressed. However, the performance is not close to the eight-cell scenario, as each cell has eight antennas that work independently. We can conclude from Figure 6a that a cooperative system with a large number of antennas has a better performance than independent systems even if the interference is suppressed.

Capacity is the second performance metric analysed in Figure 6b. In this figure, the cooperative scenario also provides the highest performance. Interestingly, we can see that in terms of capacity, a system that shares the spectrum outperforms a system with independent cells with frequency planning (eight-cells). Furthermore, when there is full inter and intra-cell interference suppression, it is possible to achieve 32% of the capacity of the cooperative system.

### 6.2. User Fairness

**Figure 7:**
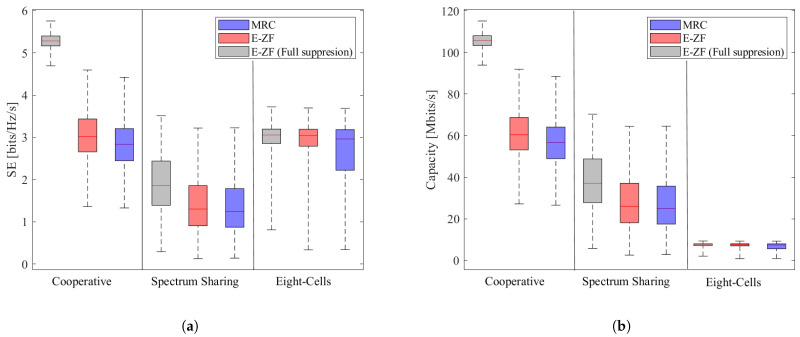
Users spectral efficiency and capacity distribution for E-ZF and MRC combining vectors. For the three system configurations: Cooperative, Spectrum Sharing and Eight-Cell, scenarios. When four interferer users are suppressed. (**a**) User spectral efficiency distribution per system. (**b**) User capacity distribution per system.

In the previous section, we discuss the performance of the scenarios in general. However, not all the users will have the same value in terms of spectral efficiency and capacity, as shown in Figure 7. This figure depicts the distribution of the performance for all the served users.

In terms of spectral efficiency, Figure 7a compares the performance for scenarios with different levels of cooperation for three types of combining vectors: E-ZF with full interference suppression, E-ZF with partial interference suppression (four users) and MRC. As expected, the mean spectral efficiency per user (red line inside each boxplot) has the highest value for a cooperative scenario with full interference suppression. Furthermore, the mean values of E-ZF with partial suppression and MRC for the cooperative and spectrum sharing cases are similar.

The fairness is estimated as the standard deviation for each boxplot presented in Table 3 and Table 4 for spectrum efficiency and capacity, respectively. In the case of spectrum efficiency, Table 3 shows that full interference suppression increases the average spectral efficiency but also significantly reduces the standard deviation and, hence, significantly improves user fairness. Regardless of the combining vector, a fully cooperative case provides better user fairness.

The distribution of the capacity between multiple users is presented in Figure 6b, and their standard deviation values in are presented Table 4. Similarly, as the spectral efficiency distribution, the capacity for a cooperative scenario under a full interference suppression provides the highest capacity with the lowest standard deviation.

### 6.3. Complex Multiplications

**Figure 8:**
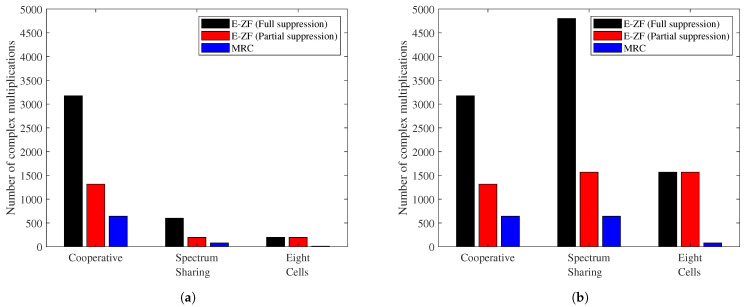
Computational cost required to estimate the different combining vectors in each analysed scenario. For E-ZF with partial suppression, only four users are considered. (**a**) Number of complex multiplications per CPU. (**b**) Total number of complex multiplications per system.

In terms of required computation capacity, Figure 8a shows the values required per each combining vector in each CPU according to the different cooperation levels. Although a fully cooperative system provides the highest performance, it also requires a higher computational cost, mainly due to the large number of antennas required to compute each signal estimate. Therefore, in the case of an E-ZF with full suppression, it requires more than 3 k complex multiplications; as anticipated, this value is reduced by a factor of two when an E-ZF with partial suppression considers only 50% of the users. In contrast, MRC represents around 20% of the E-ZF with full suppression.

Compared with a fully cooperative scenario, the computational cost of the spectrum sharing scenario is reduced by 80% for all the combining vectors, as the number of antennas used per CPU is smaller. In the case of the eight-cell scenario, the total value for E-ZF full and partial suppression represents 12.5% of the maximum value due to a reduction in the number of antennas and interferers.

As the number of CPUs differs per scenario, Figure 8b depicts a comparison of the global number of complex multiplications in the system. The spectrum sharing case requires the highest computational cost when E-ZF with full interference suppression is applied. On the other hand, with the MRC combining vector, the cooperative and spectrum sharing scenarios need the same computational cost.

## 7. Discussion

In the previous section, we analysed the spectral efficiency and capacity for the three different scenarios described in Section 5. A cooperative system where all the antennas in the system collaborate serving a set of users has many advantages: First, as a massive MIMO system, it increases the sum spectral efficiency and capacity of the system with and without interference suppression in comparison with the other scenarios. Second, when there is full intra-cell interference suppression, the spectral efficiency and capacity values almost twofold the non-interference suppression. With full intra-cell interference suppression, the spectral efficiency and capacity per user tend to be homogeneous between them. However, the performance of such systems requires a high number of complex multiplications for the centralised CPU due to the number of antennas and increases according to the number of suppressed interferers.

When the computational cost for a single CPU and synchronisation between arrays are constrained, scenarios with multiple small cells or spectrum sharing cells are an option. In this case, there is a trade-off between spectrum efficiency and capacity. On the one hand, scenarios with small cells achieve higher spectral efficiency and user fairness. On the other hand, the spectrum sharing case provides a higher capacity but does not guarantee fairness between users, and the global computational cost for full interference suppression is the highest.

It is interesting to notice that for a multi-cell system, its performance increases when there is cooperation between the different cell antennas, rather than the suppression of interference as a partial or non-cooperative system.

## 8. Conclusions

In this work, the analysis of different cooperation levels between cells and different levels of interference suppression is analysed based on a dense indoor massive MIMO experiment. Due to the flexibility of the testbed, three scenarios with different levels of cooperation are analysed. The main findings per scenario are described below.

First, a fully cooperative scenario with a centralised CPU provides the highest performance in spectral efficiency and capacity. When there is a full intra-cell interference suppression, it provides the best user fairness. Although this is considered the best-case scenario based on the previous network metrics, the centralised CPU requires a higher computational cost due to a large number of antennas when interference suppression is used, without considering a large amount of information transmitted in the back-haul due to the antenna arrays synchronisation.

Second, the spectrum sharing scenario presents the lowest spectral efficiency, even when both inter and intra-cell interference suppression is applied. However, in terms of capacity, this scenario improves its performance. Thus, it is possible to achieve 32% capacity compared to the optimal scenario with full interference suppression, with a 20% reduction of computational cost in each CPU. However, the total number of complex multiplications between all CPUs is the highest.

Third, a system with eight independent cells without synchronisation and operating in different frequencies provides, in terms of spectral efficiency, values closer to the cooperative case (without interference suppression) and requires only 12.5% of its computational cost in each CPU as the number of antennas is lower. However, in terms of capacity, this system shows an unfavourable performance.

In conclusion, the cooperation of antennas in a system provides higher spectral efficiency and capacity to all users even when there is no interference suppression. For this particular experiment, the cooperation between antennas is doable as the testbed in use has a centralised CPU (capable enough to handle the required computation cost), and the system is perfectly synchronised. However, in real scenarios, the system should address those previous constraints.

## Figures and Tables

**Table 1:** Computational complexity required per E-ZF combining vector.

Combining Vector	Complex Multiplications
Full suppression	$\left(3{\left(\mathrm{KL}\right)}^{2}+\left(\mathrm{KL}\right)\right)\frac{M}{2}+\left({\left(\mathrm{KL}\right)}^{3}-\left(\mathrm{KL}\right)\right)\frac{1}{3}$
Partial suppression	$\left(3{\left(Q+1\right)}^{2}+\left(Q+1\right)\right)\frac{M}{2}+\left({\left(Q+1\right)}^{3}-\left(Q+1\right)\right)\frac{1}{3}$

**Table 2:** Estimated parameters used per scenario based on different levels of cooperation. The range of values depends on the allocation of users in each cell.

Scenario	Number of Cells	Users per Cell	Intra-Cell Interferes	Inter-Cell Interferes
Cooperative	1	8	7	-
Spectrum Sharing	8	0–4	0–3	5–7
Eight-cell	8	0–4	0–3	-

**Table 3:** Standard deviation values for the spectrum efficiency per user.

Scenario	E-ZF (Full Suppression)	E-ZF (Partial Suppression)	MRC
Cooperative	0.1646	0.5748	0.5484
Spectrum Sharing	0.6574	0.6602	0.6259
Eight-cell	0.4621	0.6808	0.8183

**Table 4:** Standard deviation values for the capacity per user.

Scenario	E-ZF (Full Suppression)	E-ZF (Partial Suppression)	MRC
Cooperative	3.2918	11.4958	10.9682
Spectrum Sharing	13.3483	13.2049	12.5185
Eight-cells	1.1553	1.7021	2.0457

## Data Availability

Data available in a publicly accessible repository in: https://www.esat.kuleuven.be/telemic/research/NetworkedSystems/infrastructure/massive-mimo-5g (accessed on 1 March 2021).

## Abbreviations

The following abbreviations are used in this manuscript:

BS	Base Station
CPU	Central Processing Unit
D-ULA	Distributed Uniform Linear Array
E-ZF	Extended Zero-Forcing
MIMO	Multiple Input Multiple Output
MMSE	Minimum Mean Squared Ratio
MRC	Maximum-Ratio Combining
ORCA	Orchestration and Reconfiguration Control Architecture
QoS	Quality-of-Service
RZF	Regular Zero-Forcing
SIR	Signal-to-Interference Ratio
SNR	Signal-to-Interference and Noise Ratio
SNR	Signal-to-Noise Ratio
ZF	Zero-Forcing

## References

[1] Larsson E.G., Edfors O., Tufvesson F., Marzetta T.L. (2014). Massive MIMO for next generation wireless systems. IEEE Commun. Mag..

[2] Liu Z., Dai L. (2014). A Comparative Study of Downlink MIMO Cellular Networks with Co-Located and Distributed Base-Station Antennas. IEEE Trans. Wirel. Commun..

[3] Wang J., Dai L. (2015). Asymptotic rate analysis of downlink multi-user systems with co-located and distributed antennas. IEEE Trans. Wirel. Commun..

[4] Kamga G.N., Xia M., Aïssa S. (2016). Spectral-efficiency analysis of massive MIMO systems in centralized and distributed schemes. IEEE Trans. Commun..

[5] Chen C., Volskiy V., Chiumento A., der Perre L.V., Vandenbosch G.A.E., Pollin S. Exploration of User Separation Capabilities by Distributed Large Antenna Arrays. Proceedings of the 2016 IEEE Globecom Workshops (GC Wkshps).

[6] Guevara A.P., De Bast S., Pollin S. Massive MIMO: A Measurement-Based Analysis of MR Power Distribution. Proceedings of the GLOBECOM 2020—2020 IEEE Global Communications Conference.

[7] Guevara A.P., De Bast S., Pollin S. MaMIMO User Grouping Strategies: How much does it matter?. Proceedings of the 2019 53rd Asilomar Conference on Signals, Systems, and Computers.

[8] De Bast. S., Guevara A.P., Pollin S. CSI-based Positioning in Massive MIMO systems using Convolutional Neural Networks. Proceedings of the 2020 IEEE 91st Vehicular Technology Conference (VTC2020-Spring).

[9] Frisanco T., Tafertshofer P., Lurin P., Ang R. Infrastructure sharing and shared operations for mobile network operators from a deployment and operations view. Proceedings of the NOMS 2008—2008 IEEE Network Operations and Management Symposium.

[10] Rebato M., Mezzavilla M., Rangan S., Zorzi M. Resource sharing in 5G mmWave cellular networks. Proceedings of the 2016 IEEE Conference on Computer Communications Workshops (INFOCOM WKSHPS).

[11] Guevara A.P., De Bast S., Pollin S. Hardware and Spectrum Sharing for Distributed Massive MIMO. Proceedings of the 2018 52nd Asilomar Conference on Signals, Systems, and Computers.

[12] Li X., Björnson E., Larsson E.G., Zhou S., Wang J. (2017). Massive MIMO with multi-cell MMSE processing: Exploiting all pilots for interference suppression. J. Wirel. Com. Netw..

[13] Spencer Q.H., Swindlehurst A.L., Haardt M. (2004). Zero-forcing methods for downlink spatial multiplexing in multiuser MIMO channels. IEEE Trans. Signal Process..

[14] Interdonato G., Karlsson M., Bjornson E., Larsson E.G. (2020). Local partial zero-forcing precoding for cell-free massive mimo. IEEE Trans. Wirel. Commun..

[15] Marzetta T., Larsson E., Yang H., Ngo H. (2016). Fundamentals of Massive MIMO.

[16] Buzzi S., D’Andrea C., D’Elia C. User-centric cell-free massivemimo with interference cancellation and local zf downlink precoding. Proceedings of the 2018 15th International Symposium on Wireless Communication Systems (ISWCS).

[17] Veetil S.T., Kuchi K., Ganti R.K. (2015). Performance of pzf and mmse receivers in cellular networks with multi-user spatial multiplexing. IEEE Trans. Wirel. Commun..

[18] Ashikhmin A., Li L., Marzetta T.L. (2018). Interference reduction in multi-cell massive mimo systems with large-scale fading precoding. IEEE Trans. Inf. Theory.

[19] Geraci G., Garcia-Rodriguez A., Lopez-Perez D., Giordano L.G., Baracca P., Claussen H. Indoor massive mimo deployments for uniformly high wireless capacity. Proceedings of the 2018 IEEE Wireless Communications and Networking Conference (WCNC).

[20] Björnson E., Sanguinetti L., Debbah M. Massive MIMO with imperfect channel covariance information. Proceedings of the 2016 50th Asilomar Conference on Signals, Systems and Computers.

[21] Guevara A.P., Chen C., Pollin S. Partial Multi-Cell MMSE Vector Combining to Reduce Computational Cost for Massive MIMO Systems. Proceedings of the ICC 2019—2019 IEEE International Conference on Communications (ICC).

[22] Björnson E., Hoydis J., Sanguinetti L. (2017). Massive MIMO Networks: Spectral, Energy, and Hardware Efficiency. Found. Trends Signal Process..

[23] Lo T.K.Y. (1999). Maximum ratio transmission. IEEE Trans. Commun..

[24] Chen C.M., Volski V., Van der Perre L., Vandenbosch G.A., Pollin S. (2017). Finite large antenna arrays for Massive MIMO: Characterization and system impact. IEEE Trans. Antennas Propag..

[25] National Instruments (2017). 5G Massive MIMO Testbed: From Theory to Reality (White Paper). http://www.ni.com/white-paper/52382/en/.

[26] Kotzsch V., Felber C., Pollin S., Vermeulen T., Danneberg M., Bomfin R., Liu W., Moerman I., Seskar I., Nahler A. (2017). Orchestration and Reconfiguration Control Architecture, SC2: Definition of showcases. https://www.orca-project.eu/wp-content/uploads/sites/4/2017/01/ORCA_D2.1_Final.pdf.

